# The Impact of Incomplete Linkage Disequilibrium and Genetic Model Choice on the Analysis and Interpretation of Genome-wide Association Studies

**DOI:** 10.1111/j.1469-1809.2010.00579.x

**Published:** 2010-07

**Authors:** Mark M Iles

**Affiliations:** Section of Epidemiology and Biostatistics, Cancer Research UK Clinical Centre, Leeds Institute of Molecular Medicine, University of Leeds, St James's University HospitalBeckett Street, Leeds, LS9 7TF, UK

**Keywords:** mode of inheritance, genome-wide association, linkage disequilibrium, modelling

## Abstract

When conducting a genetic association study, it has previously been observed that a multiplicative risk model tends to fit better at a disease-associated marker locus than at the ungenotyped causative locus. This suggests that, while overall risk decreases as linkage disequilibrium breaks down, non-multiplicative components are more affected. This effect is investigated here, in particular the practical consequences it has on testing for trait/marker associations and the estimation of mode of inheritance and risk once an associated locus has been found. The extreme significance levels required for genome-wide association studies define a restricted range of detectable allele frequencies and effect sizes. For such parameters there is little to be gained by using a test that models the correct mode of inheritance rather than the multiplicative; thus the Cochran-Armitage trend test, which assumes a multiplicative model, is preferable to a more general model as it uses fewer degrees of freedom. Equally when estimating risk, it is likely that a multiplicative risk model will provide a good fit to the data, regardless of the underlying mode of inheritance at the true susceptibility locus. This may lead to problems in interpreting risk estimates.

## Introduction

It has been noted (Iles, 2008, Chris Spencer, personal communication) that when estimating relative risk in a genetic association study a multiplicative risk model (where the relative risk to individuals who are homozygous for the high risk allele is the square of those who are heterozygous) tends to provide a better fit to the data than a general risk model. It has been suggested (Iles, 2008) that this may be as a result of the breakdown of non-multiplicative modes of inheritance when testing a marker locus in incomplete linkage disequilibrium (LD) with the causal locus.

While interest in this issue may seem purely academic, it has important consequences both for the detection of disease-associated loci and the estimation of their effect on risk. If it were true that a multiplicative model fits just as well as the true mode of inheritance when applied to a marker locus, this would validate the use of the Cochran-Armitage trend test, which is commonly used in genome-wide association (GWA) studies. In this case, using a more general test would be wasteful as more degrees of freedom would be required, unnecessarily reducing power to detect genotype-phenotype associations. The impact of such a finding on the interpretation of risk estimation is also important – if the estimated model routinely indicates that a multiplicative risk model is correct even when it is not, this suggests that there may be systematic bias in risk estimation and the impact of a locus on disease risk may be misrepresented.

Here such effects are investigated using realistically-simulated data under a variety of underlying disease modes of inheritance and a range of marker risk models.

## Methods

We simulated two biallelic loci (e.g. single nucleotide polymorphisms or SNPs) in a case-control sample. The first locus (locus A) directly influences an individual's risk of developing a disease while the second locus (locus B) is in LD with it. The mode of inheritance at locus A is either dominant or recessive with the frequency of the high-risk allele varying between 0.01 and 0.99. The allele frequency at locus B is initially assumed to be the same as at locus A and the LD between them (measured by the squared correlation coefficient r^2^) equals 1 (complete LD), 0.8 or 0.5. The relative risk of the high-risk genotype(s) is 1.1, 1.3 or 2 and the baseline risk is assumed to be 0.05 throughout. The suitability of a multiplicative risk model at locus B was compared to fitting the true disease mode of inheritance by assuming an infinite population with equal numbers of cases and controls and fitting a logistic regression, measuring the variation captured by the model using pseudo-r^2^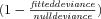. The ratio of the proportion of variation captured by fitting the true mode of inheritance (but fitted at the marker locus) and that captured by a multiplicative risk model was calculated. The closer this value was to 1, the smaller the difference between the models: ratios much greater than 1 indicate that the true mode of inheritance provides a far better fit and that a multiplicative model is unsuitable. We also calculated the sample size required for 80% power to reach a p-value of 5×10^−7^ (approximate “genome-wide significance”, (Wellcome Trust Case Control Consortium, 2007)) using logistic regression, when the two loci are in complete LD and the correct mode of inheritance is fitted at locus B (so an overestimation of true power).

In order to investigate the effect of incomplete LD on risk estimation, relative risk was estimated at locus B using both the correct mode of inheritance and a multiplicative model. Estimated relative risk to heterozygotes (relative to homozygotes for the low risk allele) was compared under the two models.

We also looked at the effect on the relative fit of the true mode of inheritance and the multiplicative model if the susceptibility allele was at a different frequency to the associated allele at the marker locus. Both dominant and recessive models were investigated, simulating as before, with GRR = 1.1, 1.3 and 2. The susceptibility allele frequency was fixed at 0.3, 0.5 or 0.7. LD was simulated between the marker and susceptibility locus such that r^2^= 0.3, 0.5 and 0.8 while the associated marker allele frequency ranged from 0 to 1. The range of allele frequencies that were possible at the marker locus was restricted, given that the two SNPs were in LD.

## Results

**Figure 1:**
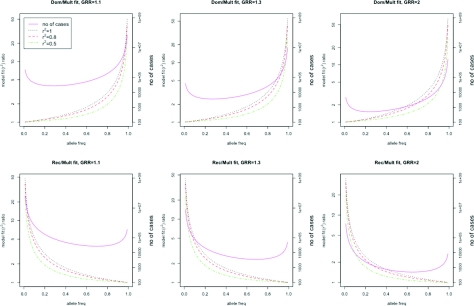
Relative fit of true mode of inheritance and multiplicative models and study power. The black (dotted), red (dashed) and green (dashed and dotted) lines show the ratio of the fit of the true mode of inheritance at the marker locus with that of a multiplicative risk model for r^2^= 1, 0.8 and 0.5 respectively. The axis for these lines is on the left hand side of each graph. The magenta (solid) line shows the number of cases (and controls) required to have 80% power to reach p = 5 × 10^−7^ if r^2^= 1. The axis for sample size is on the right hand side of each graph. The top row is when the true mode of inheritance is dominant and the bottom row recessive. The columns are for GRR = 1.1, 1.3 and 2.

The ratio of the fit of the true mode of inheritance to that of a multiplicative model was, as expected, always ≥1 (Fig. 1), indicating that a multiplicative risk model is never a better fit than the true mode of inheritance at the marker locus. For a dominant mode of inheritance, the fit of the true and multiplicative models were virtually identical for rare susceptibility alleles with the ratio increasing monotonically with allele frequency. This is because if the susceptibility allele is rare, so the high-risk homozygote will be rare, and either model will fit the heterozygote and low risk homozygote equally well. A similar pattern is seen for the recessive mode of inheritance where the true and multiplicative models fit equally well when the disease allele frequency is high and the fit of the true mode of inheritance improves relative to the multiplicative as the susceptibility allele becomes more rare. The fit of all models is unaffected by the relative risk of the disease allele. Common risk alleles for dominant modes of inheritance and rare risk alleles for recessive modes of inheritance are fitted far better by modelling the true mode of inheritance than the multiplicative. This would seem to indicate that, given allele frequency and mode of inheritance are unknown, a more general model would be better, even though this requires an extra degree of freedom. However, this doesn't account for power.

The results also demonstrate that those modes of inheritance for which the true model provides a far better fit are also those that have the lowest power to detect association (as very rare or very common variants are much harder to detect). For a dominant mode of inheritance with a genotype relative risk (GRR) of 1.1 at least 27,000 cases and controls are required to reach 80% power – far greater than is currently feasible for population-based studies. For a GRR of 1.3 at least 3,500 cases and controls are required for 80% power. If no more than 5,000 cases and controls have been collected (large for a GWA study) the susceptibility allele frequency must be between 0.12 and 0.5 to have at least 80% power to be detected. For this range of allele frequencies the ratio of the fit of modelling the true mode of inheritance to the multiplicative is no more than 1.56 (i.e. modelling the true mode of inheritance provides no more than 56% better fit) when the marker locus is in complete LD with the disease locus (equivalent to testing the disease locus); this falls to 1.25 when LD is r^2^= 0.5. Even for a GRR of 2 (far higher than most relative risks detected by GWA) if the sample size is no greater than 5000 cases and controls, and the allele frequencies are such that power is at least 80%, the ratio is 3.8 for complete LD falling to 2.2 for r^2^= 0.5. If the sample size is 2000 cases and controls (common for GWA) the ratio is 2.5 for complete LD falling to 1.7 for r^2^= 0.5. While these figures may sound big, these are quite extreme examples, the median observed relative risk (Iles, 2008) is only 1.25: for 80% power this would require at least 4950 cases and controls for a susceptibility allele frequency of 0.27 (larger numbers for other frequencies) giving only a 20% improvement in fit by modelling the true dominant mode of inheritance over the multiplicative for a marker in complete LD dropping to 9% when r^2^ between markers is 0.5. Furthermore, as well as a more general model requiring (as has been mentioned) extra degrees of freedom, dominant and recessive modes of inheritance are the most extreme examples of risk increasing monotonically with number of high risk alleles carried: if less extreme modes of inheritance were simulated, the multiplicative risk model would fit better. If the underlying mode of inheritance were multiplicative, then a multiplicative model would provide the optimal fit!

Differences in allele frequencies between the two loci made little difference to the ratio of the fit of the true and multiplicative risk models. For r^2^= 0.8, the possible range of allele frequencies at the marker locus was so small that the ratio of the fit of modelling the true mode of inheritance with the fit of the multiplicative model never varied by more than 0.15% from the value when the alleles have the same frequency. For r^2^= 0.5 or 0.3 there was more variation but the ratio of the fits for r^2^= 0.5 (Supp. Fig. 1) decreased on average by 1% and for r^2^= 0.3 (Supp. Fig. 2) decreased by 3% when allele frequency at the marker locus is varied compared to being the same as at the susceptibility locus. On average the ratio of the true and multiplicative models tended to fall slightly if allele frequencies differed, suggesting that the fit of the multiplicative model improved slightly relative to modelling the true mode of inheritance.

**Figure 2:**
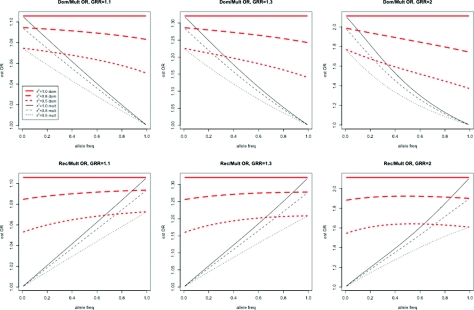
Estimated heterozygous risk when fitting true mode of inheritance and multiplicative models. The estimated heterozygous relative risk when fitting the correct mode of inheritance (red thicker lines) and when a multiplicative risk model is applied (black thinner lines). LD between the susceptibility and marker loci is simulated to be 1 (solid lines), 0.8 (dashed lines) and 0.5 (dotted lines). The top row is when the true mode of inheritance is dominant and the bottom row recessive. The columns are for GRR = 1.1, 1.3 and 2.

Our results on estimation of relative risk (Fig. 2) show that, as expected, the multiplicative model never produces heterozygote risk estimates closer to the true value than fitting the correct mode of inheritance, but that the two models produce the same results for a dominant mode of inheritance when the disease allele frequency is low and for a recessive mode of inheritance when the disease allele frequency is high. As frequencies move away from these values the risk estimates diverge. For instance, for GRR = 1.3 and a sample size of 5000 cases and controls, for a power of 80%, the disease allele frequency must be <0.5 (assuming r^2^= 1 between marker and disease locus). For these values if r^2^= 1 the heterozygous risk estimate when fitting the true mode of inheritance is 1.32 (greater than 1.3 because an odds ratio) and under the multiplicative is ≥1.15 (average of 1.23). For r^2^= 0.8 the heterozygous risk estimate when fitting the true mode of inheritance is ≥1.27 (average of 1.28) and under the multiplicative is ≥1.13 (average of 1.21). For r^2^= 0.5 the heterozygous risk estimate when fitting the true mode of inheritance is ≥1.19 (average of 1.21) and under the multiplicative is ≥1.10 (average of 1.16). Thus when the associated locus is in incomplete LD with the causative locus, using a multiplicative risk model is likely to slightly underestimate compared to modelling the true mode of inheritance.

## Discussion

The importance of these results is twofold, impacting both on the analysis of genome-wide association studies and on the interpretation of the results.

In terms of analysis the results suggest that while fitting a multiplicative model will never be better than fitting the true mode of inheritance, when the study is well-powered to find an associated locus, investigators will lose little by fitting a multiplicative model such as the Cochran-Armitage trend test.

In terms of risk estimation, for well-powered studies a multiplicative model is likely to provide a good fit to the data, potentially leading investigators to falsely conclude that the true mode of inheritance is multiplicative. Furthermore, estimating risk from an associated marker locus rather than the susceptibility locus will always lead to an underestimation of true risk with further loss resulting from fitting a multiplicative model rather than fitting the true mode of inheritance. While not inconsequential, the further underestimation caused by fitting a multiplicative model at an associated locus appears to be of a similar order to the underestimation initially caused by examining an associated locus rather than the causative locus. At the same time risk is likely to be inflated due to the so-called “Winner's curse” (Göring et al., 2001; Zöllner and Pritchard, 2007).

While the models simulated here have been made as realistic as is feasible while retaining generality, there are several limitations that will tend to favour the fit of the true mode of inheritance over the multiplicative:

- Our power calculation assumes complete LD and the correct mode of inheritance being known. Both of these are unlikely, thus power would be lower and the range of detectable allele frequencies even more restricted.
- Fit is compared here between a multiplicative model and modelling the true mode of inheritance. But the latter is unknown. Thus a more general model would be fitted requiring extra degrees of freedom, resulting in loss of power.
- The mode of inheritance at the susceptibility locus may not be completely dominant or recessive (in classical genetic terms the dominance component may be smaller relative to the additive component) and so closer to a multiplicative mode of inheritance. Again this would result in the multiplicative model fitting better. The mode of inheritance may even be multiplicative.

In a genome-wide association study, there may be several loci that are potentially detectable. If there are five loci, power to detect at least one with 80% power requires just under 30% power to detect each one separately. However, judging by results so far it is unlikely that there are many unknown common loci with large effects remaining (Easton et al., 2007; Iles, 2008). Most major traits have already been the subject of at least one large GWA study, so further findings will be reliant on larger sample sizes and better SNP coverage, both of which are liable to only incrementally increase power.

Overall these results indicate that, due to incomplete LD, marker loci are likely to be well-fitted by a simple multiplicative model. Thus for GWA a Cochran-Armitage trend test should be adequate. When estimating risk at a disease-associated locus a multiplicative risk model is likely to fit well, whatever the mode of inheritance at the true susceptibility locus and potentially lead to a slight underestimation of risk.

## Supporting Information

**Figure S1.** Relative fit of true mode of inheritance and multiplicative models when allele frequencies differ, r^2^ = 0.5.

**Figure S2.** Relative fit of true mode of inheritance and multiplicative models when allele frequencies differ, r^2^ = 0.3.

**Figure S3.** Comparison of the association power using a multiplicative (Mult), dominant (Dom) or general 2d.f. (ChiSq) test of association.

As a service to our authors and readers, this journal provides supporting information supplied by the authors. Such materials are peer-reviewed and may be re-organised for online delivery, but are not copy-edited or typeset. Technical support issues arising from supporting information (other than missing files) should be addressed to the authors.
