# Supplementary material for: The Impact of Incomplete Linkage Disequilibrium and Genetic Model Choice on the Analysis and Interpretation of Genome-wide Association Studies
Source: Ann Hum Genet. 2010 Jul;74(4):375–9. doi: 10.1111/j.1469-1809.2010.00579.x (PMC2905613; doi:10.1111/j.1469-1809.2010.00579.x)
Supplement: Supplementary file 1 [file ahg0074-0375-SD1.doc]

SUPPLEMENTARY FIGURE LEGENDS

Supplementary Figure 1. Relative fit of true mode of inheritance and multiplicative models when allele frequencies differ, r^2^=0.5.

The black, red and green lines show the ratio of the fit of the true mode of inheritance at the marker locus with that of a multiplicative risk model when the susceptibility allele frequency is 0.7, 0.5 and 0.3, respectively for a range of allele frequencies at the marker locus and LD between the two loci of r^2^=0.5. The top row is when the true mode of inheritance is dominant and the bottom row recessive. The columns are for GRR=1.1, 1.3 and 2.

Supplementary Figure 2. Relative fit of true mode of inheritance and multiplicative models when allele frequencies differ, r^2^=0.3.

The black, red and green lines show the ratio of the fit of the true mode of inheritance at the marker locus with that of a multiplicative risk model when the susceptibility allele frequency is 0.7, 0.5 and 0.3, respectively for a range of allele frequencies at the marker locus and LD between the two loci of r^2^=0.3. The top row is when the true mode of inheritance is dominant and the bottom row recessive. The columns are for GRR=1.1, 1.3 and 2.

Supplementary Figure 3. Comparison of the association power using a multiplicative (Mult), dominant (Dom) or general 2d.f. (ChiSq) test of association.

Sample sizes are calculate to give 80% power for the dominant test (up to a maximum sample size of 10,000 cases and controls). GRR for heterozygotes for the causative allele=1.3 (close to the median observed: Iles, 2008). GRR for homozygotes for the causative allele ranges from 1.3 (dominant) to 1.69 (multiplicative) along the x-axis. LD between the marker and causative locus =1 (first column), 0.8 (second column) or 0.5 (third column). Causative allele frequency =0.5 (first row), 0.25 (second row) or 0.1 (third row). Power calculation based on 5000 simulations.


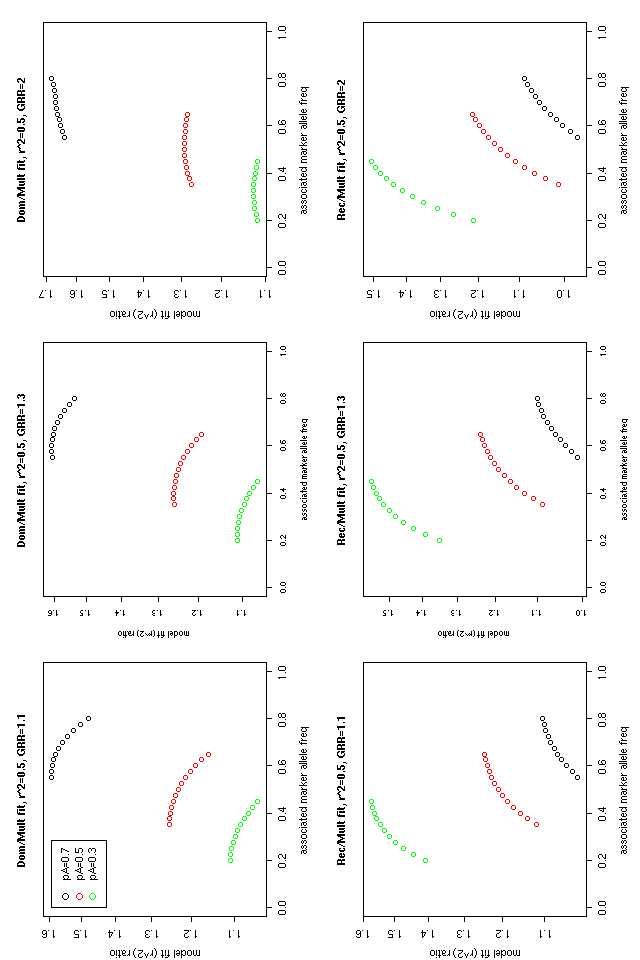


Supplementary Figure 1


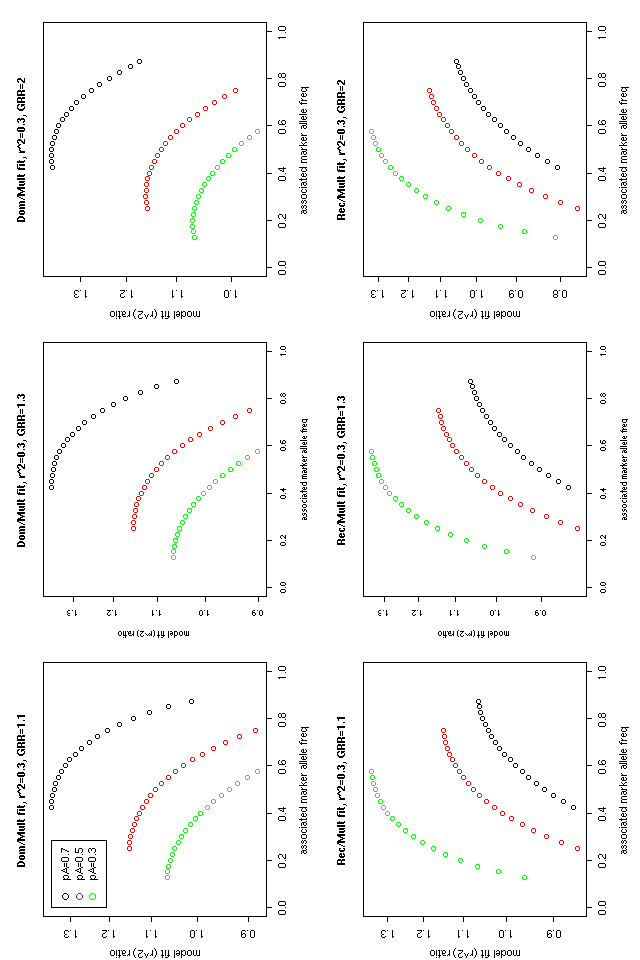


Supplementary Figure 2


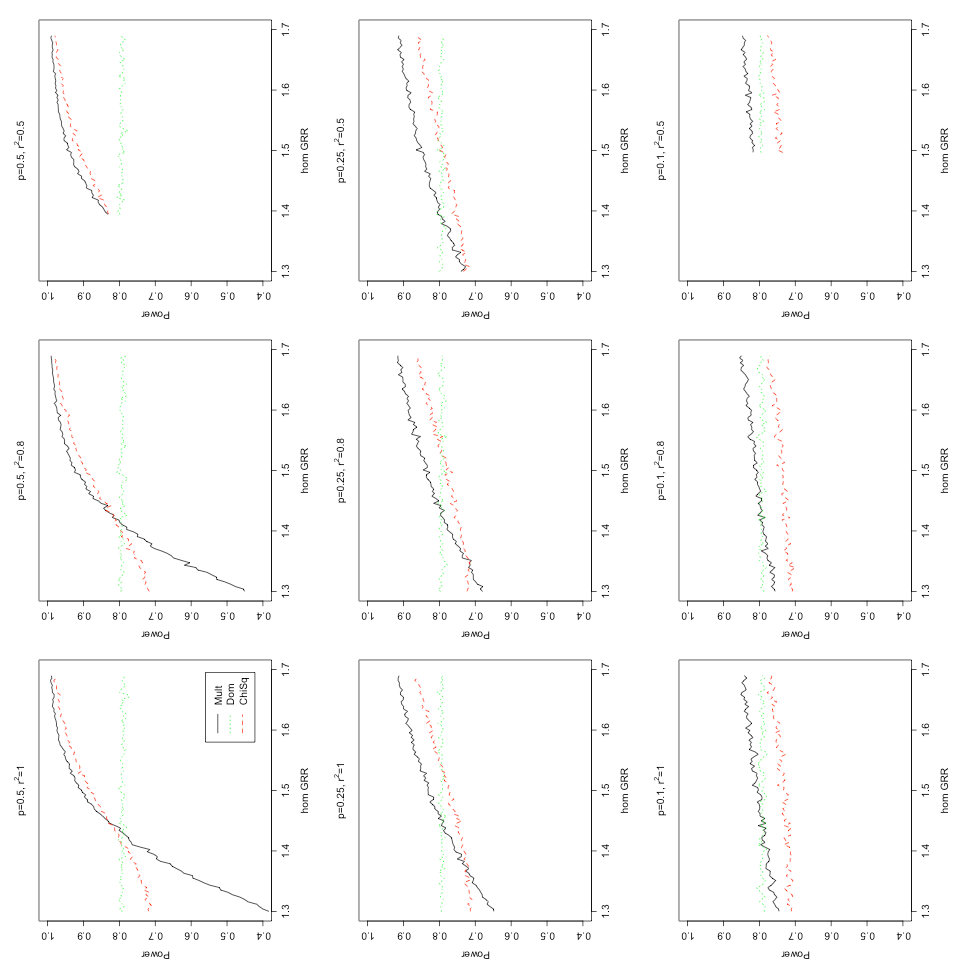


Supplementary Figure 3
